# Structural and Electronic Effects at the Interface between Transition Metal Dichalcogenide Monolayers (MoS_2_, WSe_2_, and Their Lateral Heterojunctions) and Liquid Water

**DOI:** 10.3390/ijms231911926

**Published:** 2022-10-07

**Authors:** Zhen Cao, Moussab Harb, Sergey M. Kozlov, Luigi Cavallo

**Affiliations:** 1 Kaust Catalysis Center (KCC), Physical Sciences and Engineering Division (PSE), King Abdullah University of Science and Technology (KAUST), Thuwal 23955-6900, Saudi Arabia; 2 Department of Chemical and Biomolecular Engineering, Faculty of Engineering, National University of Singapore, Singapore 119260, Singapore

**Keywords:** 2D materials, transition metal dichalcogenides, water 2D materials interface, density functional theory, molecular dynamics

## Abstract

Transition metal dichalcogenides (TMDCs) can be used as optical energy conversion materials to catalyze the water splitting reaction. A good catalytical performance requires: (i) well-matched semiconductor bandgaps and water redox potential for fluent energy transfer; and (ii) optimal orientation of the water molecules at the interface for kinetically fast chemical reactions. Interactions at the solid–liquid interface can have an important impact on these two factors; most theoretical studies have employed semiconductor-in-vacuum models. In this work, we explored the interface formed by liquid water and different types of TMDCs monolayers (MoS_2_, WSe_2_, and their lateral heterojunctions), using a combined molecular dynamics (MD) and density functional theory (DFT) approach. The strong interactions between water and these semiconductors confined the adsorbed water layer presenting structural patterns, with the water molecules well connected to the bulk water through the hydrogen bonding network. Structural fluctuations in the metal chalcogenide bonds during the MD simulations resulted in a 0.2 eV reduction of the band gap of the TMDCs. The results suggest that when designing new TMDC semiconductors, both the surface hydrophobicity and the variation of the bandgaps originating from the water-semiconductor interface, need to be considered.

## 1. Introduction

Two dimensional semiconductors could be good candidates for photoelectrochemical hydrogen evolution reactions (HER) [1,2,3,4,5,6,7,8,9]. In practice, the semiconductor works as a heterogeneous catalyst to convert solar energy and water to form sustainable hydrogen. This process requires that the band gaps of these semiconductors well fit the visible region of solar light [10]. Additionally, the conducting band minimum (CBM) and the valence band maximum (VBM) must be well aligned to straddle over the reduction and oxidation potentials of water [11]. Within this framework, transition metal dichalcogenides (TMDCs) and their lateral heterojunctions can well meet the above requirement, and have attracted broad attention due to their ideal electronic structures [1,3,4,5,9,11,12,13,14,15,16,17,18]. Meanwhile, many theoretical simulations have been performed targeting the tunable bandgaps [19] and the energetics of the HERs on TMDCs [20,21]. However, these simulations were performed under a gas phase condition, while the HER reactions occurred in aqueous solution.

Within the pH-neutral environment usually required in applications of electrochemical processes [22,23], interaction of water with solids can affect the network of H-bonds within water [24,25,26,27] and result in water adopting a layered structure at the interfacial region denser than in bulk liquid. This dense layer of water molecules can further induce pronounced solvation shells in the liquid phase. The resulting altered structure of liquid water near the semiconductor surface has been suggested to impact the thermodynamics and kinetics of surface and near-surface reactions [28,29,30], and to also modify the atomic and electronic structure of the semiconductor [29]. For example, it has been shown that the band edges of hydrophobic silicon surfaces can be shifted by approximately 0.5 eV when exposed to water, evidencing the ability of water to affect the properties of the semiconductor several atomic layers away from the interface. In turn, this finding suggests that monolayer (or 2D) materials could also be affected by interaction with water due to the subnanometer thickness and extremely high “surface-to-bulk” ratio.

Considering TMDCs in water scenario, the hydrophobic character of TMDCs [31,32] can lead to a denser layer of water staying at the interface. These interfacial water molecules further induce a pronounced structure of water impacting the double layer region where the catalyzed HER occurs. Unfortunately, the experimental characterization of the double layer region is extremely challenging [33] and, thus, computational modeling becomes a privileged approach for investigation [28].

Considering the numerous unanswered questions relating to the behavior of TMDCs in water, we performed a density functional theory (DFT) study of liquid water–TMDCs interfaces of pure MoS_2_ and WSe_2_, and of the LHJs of these two materials presenting armchair and zig-zag geometries, see Figure S1. The more realistic description of water–TMDCs interfaces is achieved by DFT molecular dynamics (MD) simulations augmented by detailed characterization of the electronic structure of the TMDCs in selected snapshots from the DFT-MD trajectories. The aim of this study was to shed light on the liquid–solid interface for MoS_2_ and WSe_2_ monolayers, and their LHJs, with regard to: (i) the structure of water under the confinement originating from the TMDCs monolayers, with possible implications on catalytic behavior; and (ii) how water and dynamics affect the electronic properties of TMDCs monolayers.

## 2. Results and Discussions

### 2.1. Water Structure at the TMDCs-Water Interface

**Figure 1:**
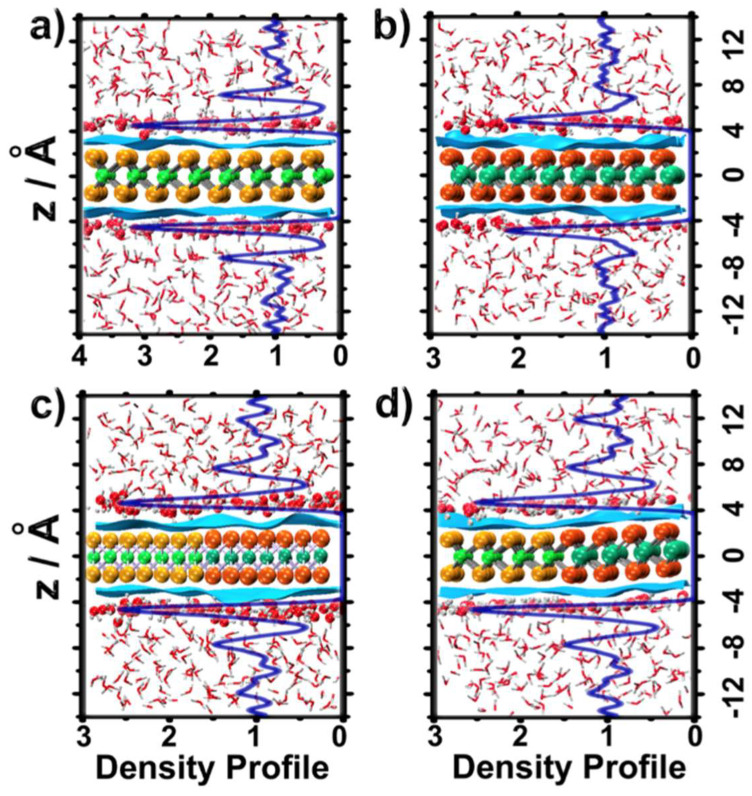
DFT-MD density profiles of water oxygen weighted by the density of bulk water, ρ_O_(z), along *z*, which is the direction perpendicular to the interfacial region, for different systems: (**a**) MoS_2_, (**b**) WSe_2_, (**c**) armchair LHJ, and (**d**) zigzag LHJ. The inserted blue surfaces are the instantaneous surfaces of liquid water. Atomic positions and the water surfaces correspond to the last frame of the DFT-MD simulations.

We start the discussion by analyzing the density profiles of the water O atoms, ρ_O_(z), along the direction perpendicular to the TMDC surface (Figure 1). We first noticed that all the density profiles approached ρ_O_(z) = 1 (normalized by bulk water density) at a large distance from the TMDCs’ surface (|z| ≥ |0 Å), with a consistent double layer thickness of ≈10 Å [34]. All the density profiles showed a high peak, ρ_O_(z) ≈ 2–3, at ≈2.5 Å from the surface (|z| ≈ 5–6 Å), indicating a high-density structure of the first water layer.

**Figure 2:**
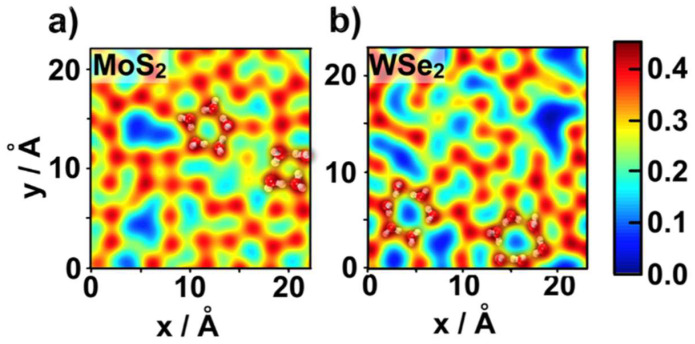
Probability maps, $\phi_{w}\left(x,y\right)$, of finding ad-layer water molecules on top: (**a**) MoS_2_, (**b**) WSe_2_. Ad-layer water molecules correspond to water molecules within the first minima (|z| ≈ 5 Å) of the water density profiles ρ_O_(z), shown in Figure 1.

Comparison of the density profiles in the presence of MoS_2_ and WSe_2_ (Figure 2a,b) indicated a higher first peak for MoS_2_, ρ_O_(z) ≈ 3, compared with WSe_2_, ρ_O_(z) ≈ 2. The heights of the first peaks in the density profiles of the water–LHJ systems (Figure 1c,d) were located between those of the pure water–MoS_2_ and water–WSe_2_ systems. For simplicity, this high-density water layer will be called ad-layer, in the rest of this paper.

To visualize water morphology in the ad-layer, the instantaneous surface [35] of liquid water at the end of the DFT-MD trajectory was inserted in Figure 1. The wavy character of the surfaces indicated that the distribution of water molecules in the ad-layer was not uniform. Integrated over time, these water molecules constructed the first peak of the density profiles inserted in Figure 1.

To investigate the structure of water molecules at the interface, we projected the interfacial water density on top of a given position of the TMDC surface to calculate $\phi_{w}\left(x,y\right)$. This function (detailed in Supplementary Materials) quantified the probability of finding oxygen atoms of the interfacial water molecules above a given position over the TMDC surface (reddish color spots in Figure 2) [22]. The numerous blue patches in Figure 2b are consistent with the relatively lower first peak in the density profile ρ_O_(z) of WSe_2_, compared with that of MoS_2_.

The Figure 2 probability maps also highlight structural motifs adopted by water molecules in the ad-layer. Since the S-S and Se-Se distances (3.18 and 3.32 Å) were comparable with the distance between two water oxygen atoms connected by a hydrogen bond (2.5–3.4 Å in liquid water), water molecules in the ad-layer formed a stable intra-layer hydrogen bonding network (HBN) of ring-like structures matching the periodicity of the TMDCs surface [22,30,36]. A longer Se-Se distance can result in the formation of larger ring-like water structures on WSe_2_ (e.g., the six-member ring marked in Figure 2b), reminiscent of water adsorbed on the surface of a variety of metals [25,27,33]. Differently, smaller ring-like structures composed of four or five water molecules mainly formed on top of MoS_2_ (Figure 2a). The TMDC induced less ordered water topology compared with water on top of the (111) surfaces of platinum and palladium [30,37], because the hydrophobicity of TMDCs led to a weaker attraction on ad-layer water molecules, and this water layer could not fully reflect the topology of TMDC surface. The blue patterns (empty sites) may also serve as additional active sites for catalyzed HER reactions.

The smaller atomic radius of S compared with Se, resulted in closer proximity of the interfacial water molecules to the MoS_2_ surface relative to the WSe_2_ surface. This is captured by the map of Figure S4b, obtained with a cut-off for the definition of water molecules in the ad-layer, reduced by 15%. In this case, reddish patches corresponding to adsorbed water molecules can only be seen above the MoS_2_ side of the LHJ (Figure S4b). Considering that the electron transfer rate was inversely proportional to the distance between electrode and electrolyte, [38] this closer interfacial water layer may indicate that MoS_2_ could serve as a better HER catalyst.

**Figure 3:**
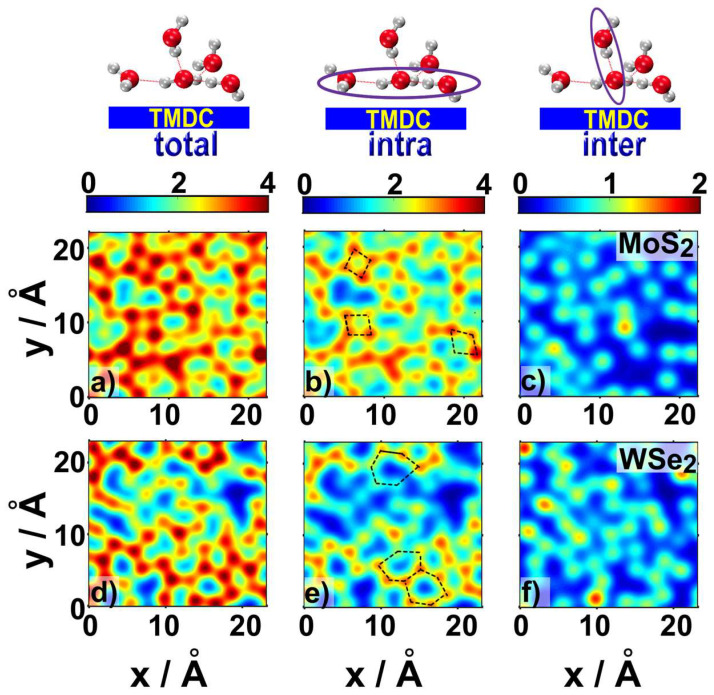
The hydrogen bonding networks above TMDCs were evaluated in three ways: the total number of H-bonds for ad-layer water molecules, n_HB_(x,y), above MoS_2_ (**a**), and WSe_2_ (**d**). The total number of H-bonds were decomposed into H-bonds formed by waters within the ad-layer (**b**,**e**), and H-bonds between ad-layer water molecules and water molecules in the above layer (**c**,**f**). Some ring structures are marked in (**b**,**e**).

As the HBN is critical for the liquid water structure [39], we investigated the average number of H-bonds formed by interfacial water molecules on top of the TMDC surface, n_HB_(x,y). As demonstrated in Figure 3a,d (Figure S5 for LHJs), each adsorbed water molecule formed ~4 H-bonds during the MD trajectory, independent of the tendency of the specific TMDC to promote formation of four to six-membered water rings [27].

**Figure 4:**
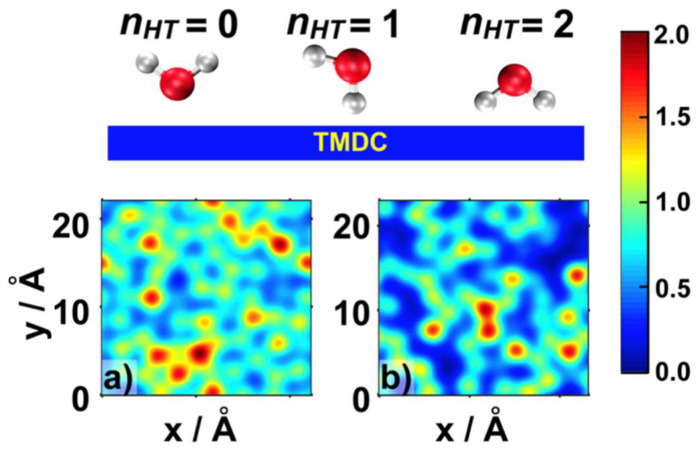
Orientation of water molecules above MoS_2_ (**a**), WSe_2_ (**b**).

The total average number of H-bonds formed by water molecules in the ad-layer, n_HB_(x,y), was decomposed into two parts: (i) H-bonds formed between water molecules within the ad-layer (middle column in Figure 4 and Figure S5); and (ii) H-bonds formed between water molecules in the ad-layer and water molecules above them (right column in Figure 4 and Figure S5). The similarity between the maps in the left and middle columns of Figure 4 indicated that most of the H-bonds formed by the interfacial water molecules were formed within the ad-layer. Nonetheless, the yellow-reddish spots in the maps in the right column of Figure 4 indicated that on average, at least one H-bond could be formed between interfacial water molecules and water molecules in the second layer above the TMDC surface. Therefore, the HBN of water molecules in the ad-layer was well connected to the HBN of water molecules in the bulk.

Water molecules in the ad-layer of WSe_2_ had a better connection with water molecules in the second layer (0.97 ± 0.06 on MoS_2_ vs. 1.24 ± 0.09 on WSe_2_). As was shown for other liquid water–solid interfaces [22,27,30,40], these H-bonds could facilitate proton transfer to the surface via the Grotthuss mechanism, which could be beneficial for electrochemical or photocatalytic applications.

Thereafter, we focused on the connection between hydrogen atoms of the interfacial water molecules, since both experimental and theoretical research demonstrate that pH-neutral HER may start from H_2_O + e^−^ + substrate -> H_ad_-substrate + OH^−^ [41,42]. To investigate the molecular orientation of adsorbed water molecules, we plotted the average number of O-H bonds of interfacial water molecules pointing towards the TMDC surface, n_HT_(x,y).

**Figure 5:**
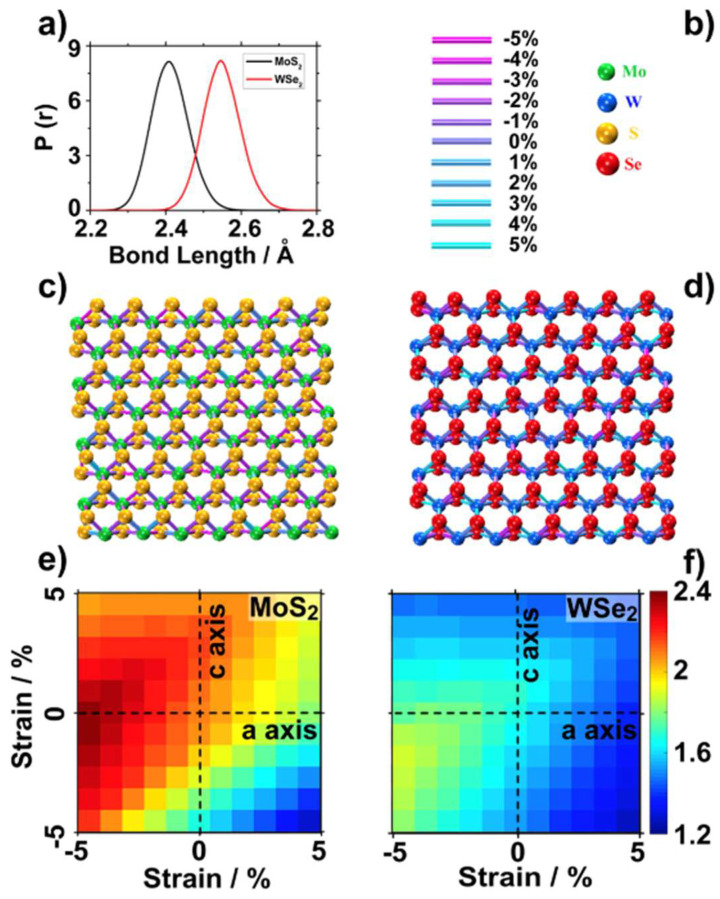
(**a**) Distribution of the Mo-S bond length and W-Se bond length throughout the DFT-MD trajectory. (**b**) Color map for stretched or compressed bond length and the color code of atoms. Snapshots of (**c**) MoS_2_, and (**d**) WSe_2_, monolayers obtained at the end of DFT-MD simulation. Water molecules are not shown, and all the bonds are colored according to (**b**). Band gap variation of (**e**) MoS_2_, and (**f**) WSe_2_, related to the strain along the a-axis and c-axis.

Since both the S and Se atoms in the TMDCs were negatively charged, and the TMDCs presented a hydrophobic feature, [31,32] interfacial water molecules tended to have at least one hydrogen atom pointing toward the TMDC surfaces. This result was also consistent with the water O/H density profiles (Figure S3), showing that a fraction of the O-H bonds pointed toward the TMDC surface. Such orientation could facilitate supply of H to the TMDC without additional re-orientational motion, boosting their electrochemical performance [43]. Comparison between MoS_2_ and WSe_2_ indicated that interfacial water molecules on top of MoS_2_ had a higher number of H atoms pointing towards the TMDC surface (Figure 5a,b).

### 2.2. Electronic Properties of Wetted TMDCs

We started by calculating the distribution of Mo-S and W-Se bond lengths throughout the DFT-MD trajectory. The instantaneous bond distances from the MD simulations were described by a Gaussian distribution centered on the DFT optimized bond lengths, with deviations of up to 5% (Figure 5a). Figure 5c,d present two snapshots of the MoS_2_ and WSe_2_ monolayers with chemical bonds colored according to their length.

As it is well accepted that the bandgaps of TMDCs can be tuned by introducing constraints [44,45], and that these structural fluctuations in bond lengths can have an impact on the TMDCs performance, we analyzed the variation in bandgaps by deforming the optimized TMDC cell parameters. For example, the uniform deformation of MoS_2_ and WSe_2_ cell parameters by −5%/+5% changed the bandgaps from 2.07 and 1.65 eV, to 2.38/1.28 eV and 1.89/1.27 eV, respectively (Figure 5e,f). This degree of bandgap variation is not acceptable for solar energy applications. Different from this extreme calculation condition, the instantaneous dynamics of the chemical bonds in a TMDC-water system is not uniform. To explore the more realistic bandgap variation due to the combined effect of the instantaneous deformation of all the metal chalcogenide bonds, we averaged the band gap and alignment of eight snapshots extracted from the last 4 ps of the MD simulations, using the hybrid HSE06 functional, including spin-orbital coupling [46].

In general, the band gaps became smaller for both TMDCs. The contraction in the band gap was larger for MoS_2_ (0.16 eV), from 2.07 eV in the structure optimized in vacuum, to 1.91 eV averaged over the eight snapshots from the DFT-MD simulations; whereas for WSe_2_, it reduced only from 1.65 eV to 1.56 eV (0.09 eV). The small variation in the band gap of the two TMDCs under dynamic conditions could be related to the anisotropic shape of the maps shown in Figure 5e, with reduction in the band gap due to strained metal chalcogenide bonds compensated by enlargements due to compressed bonds. The larger variation in the band gap of MoS_2_ relative to WSe_2_ could be related to the large impact of the unit cell deformation on the band gap of MoS_2_ (compare the two maps in Figure 5e,f). On the other hand, interfacial water under dynamic conditions did not significantly modify the electronic structure of LHJs (Figure S7).

**Figure 6:**
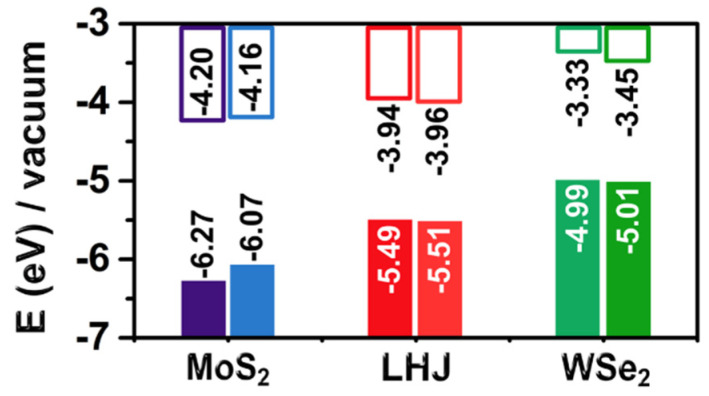
Aligned band gaps for MoS_2_, WSe_2_ and armchair or zig-zag types of LHJs toward the vacuum level of electronic potentials for both DFT-optimized structures (left columns) and DFT-MD snapshots (right columns).

Whereas a previous study of the electronic properties of silicon with functionalized hydrophobic surfaces concluded that structural deformation from dynamics did not alter the band gap of silicon [29], we observed a clear effect of dynamics on the band gap of monolayer TMDCs. This may be rationalized considering that larger structural deformations could be expected under dynamic conditions for high surface to bulk ratio materials, such as monolayer TMDCs. Further, the calculated electronic levels were aligned to the vacuum level for both the structures optimized in vacuum, and the structures from the DFT-MD simulations. Analysis of Figure 6 indicates that structural fluctuations introduced by the dynamics shifted the valence band maximum (VBM) of MoS_2_ by 0.2 eV, and the conduction band minimum (CBM) of WSe_2_ by 0.12 eV.

Next, we evaluated the impact of the water molecules on the electronic structure of the TMDCs. To this end, we compared the average bandgap and alignment of the eight structures from the MD simulations in the absence, and in the presence, of water. Due to the computational cost, this test was performed at the PBE level and for MoS_2_ only, as the TMDC was most affected by dynamics. According to calculations, the band gap of MoS_2_ was practically unaffected by the presence of water, with an average difference of only 0.02 eV on the eight frames. As for the band alignment, an average small shift of only 0.03 eV (see the Supplementary Materials for details) was calculated, indicating the negligible capability of water to directly perturb the electronic structure of the TMDC. The results indicated that the band alignment of TMDCS, even though affected by the instantaneous dynamics, could well fit the requirements for photochemical catalysts [3]. This is different to what has been reported for functionalized hydrophobic surfaces of silicon, where water was demonstrated to shift the silicon band edges by as much as 0.5 eV [29]. This can be rationalized considering that the examined functionalized silicon surfaces have a surface dipole moment allowing electrostatic interaction with water, whereas monolayer TMDCs can be considered apolar. Since we found no impact of water on both the band gap and alignment of MoS_2_, we assumed this conclusion also holds for WSe_2_ and the LHJs.

Finally, we computed the UV-visible optical absorption spectra of the TMDCs by averaging results on eight frames from the MD simulations (Figure S8) because, experimentally, light absorption can be differently modified within different solutions. In general, configurations obtained from the MD simulations demonstrated a weaker absorption at shorter wavelength region (~350 nm) and produced a shoulder in the long wavelength region (~600 nm). The latter could be rationalized by the decreased symmetry of the TMDCs structure in contact with water.

## 3. Simulation Methods

### 3.1. Empirical Force Field Development

The interactions between semiconductor and water molecules were decomposed into pairwise Coulomb interactions and vdW interactions, as follows:

(1) $$E=\sum_{i}\sum_{j>i}\left\{\frac{1}{4\pi \varepsilon_{0}}\frac{q_{i}q_{j}}{r_{ij}}+4\varepsilon_{ij}\left[{\left(\frac{\sigma_{ij}}{r_{ij}}\right)}^{12}-{\left(\frac{\sigma_{ij}}{r_{ij}}\right)}^{6}\right]\right\}$$

where *q_i_* and *q_j_* are partial charges on the *i*-th and *j*-th atom, and *ε*_0_ is the dielectric constant in vacuum.

The DFT energy was scanned as a function of distance between the water oxygen and the MoS_2_ or WSe_2_ surface, respectively, as shown in Figure S1a. The total energy was scanned as a function of distances between the semiconductor surfaces, ranging from 1.57 Å to 20.97 Å, with a 0.2-Å step-size, to generate 108 data points. Similarly, the water to WSe_2_ distances were scanned, ranging from 1.97 Å to 22.77 Å, with a 0.2-Å step-size, to generate another 108 data points. The calculations were performed using the PBE level of density functional theory through the CP2K package [47]. The DZVP-MOLOPT-SR-GTH pseudo-potential was used to describe the Mo, W, S, Se; and the DZVP-MOLOPT-GTH pseudo-potential was used to describe the electronic properties of O and H [48]. The third generation of Grimme’s dispersion correction [49] was used to describe the vdW interactions. Since we only cared about the relative energy difference between different configurations, the total energy at the longest distance was set to 0. The partial charges for water oxygen and hydrogen atoms were taken from the SPC/Fw water molecules [50]. The partial charges for Mo, W, S, and Se on the slab were fitted using the RESP method [51] implemented into the CP2K package. After removing the Coulomb interactions between the water molecules and the TMDC slabs, the genetic algorithm was used to fit the rest of the energy, which was attributed to the vdW interactions. Similar to previous studies [36,52], the vdW interactions between water molecules were coarse-grained onto the water oxygen. The fitted interaction energy is shown in Figure S1b, and our empirical model well reproduced the DFT-calculated interaction energies. The fitted parameters used in the classical molecular dynamics simulations are reported in Table S1.

### 3.2. Classical Molecular Dynamics Simulations

To efficiently sample the systems, we started from a classical molecular dynamics (MD) simulation with our empirical model, to generate good initial configurations for the subsequent DFT-MD simulations. Firstly, four systems, namely, water–MoS_2_, water–WSe_2_, water–armchair LHJ, and water–zigzag LHJ, were constructed using the optimized semiconductor monolayer together with one thousand water molecules. Five hundred water molecules were placed on each side of the semiconductor surfaces (as shown in Figure S2a), while a much longer box (z = 70 angstrom) was constructed to generate both water–semiconductor and water–vacuum interfaces. This build-up was implemented to allow water molecules to fully relax and obtain the natural density of bulk water. The four systems were pre-equilibrated in NVT ensemble at 300 K employing the Nose–Hoover thermostat for 40 ns with a 1-fs time step using the LAMMPS simulation package [36]. Since our target was to relax the water structure, the TMDCs were treated as a rigid body utilizing SHAKE algorithm to constrain bonds [53] as in the optimized structure. The last 30 ns trajectory was used to calculate the density profile of the water molecules. This density profile was weighted by the bulk density of water, where the value of 1 indicated that the properties of those water molecules were approaching bulk water. The results are shown in Figure S2b. A box with a total length of 28 angstrom, where the density profile of water was approaching bulk, along the z direction, was carved from the previous large box. Then, the new system was re-equilibrated for another 20 ns under the same simulation conditions. The last frame of each simulation trajectory was stored for the later DFT-MD simulation.

### 3.3. Density Functional Theory Molecular Dynamics (DFT-MD) Simulations

Starting from the configuration obtained from the classical MD simulation, DFT-MD simulations were performed in the NVT ensemble at 300 K using the CP2K simulation package [47] and the PBE [54] exchange-correlation functional. The DZVP-MOLOPT-SR-GTH pseudo-potential was used to describe the Mo, W, S, Se; and the DZVP-MOLOPT-GTH pseudo-potential was used to describe the electronic properties of O and H [48]. The third generation of Grimme’s dispersion [49] correction was used to describe the vdW interactions. Parinello’s CSVR thermostat [55] was used to control the temperature. The simulations were performed for 5 ps with a 0.5 fs time step for each system, and the last 4 ps trajectory was recorded every step, for the following analysis.

### 3.4. Density Functional Theory Geometry Optimizations

The optimizations were performed for 7 × 8 units for the MoS_2_ monolayer, WSe_2_ monolayer, and armchair/zigzag prototypical TMDCs, through the VASP package [56,57,58,59]. In this study, we performed the calculations using the PBE functional and the projected augmented plane wave method. The default cut-off energy of 280 eV was employed, the convergence criterion for the SCF cycles was set to 0.001 meV per super lattice, and the criteria for the Hellmann–Feynman forces on each atom was set to 0.001 eV/Å, following the setup we used before, which could well reproduce the experimental results.

### 3.5. Electronic Property Calculations

The calculations were performed at the HSE06 level [60] of density functional theory with spin orbit coupling [61]. The configurations were obtained from the MD simulation trajectory. The electrons were described using the 400 eV plane wave implemented in the VASP package. The band gaps were aligned in two ways to extract different information. In the first way, the band gaps were aligned to the vacuum energy (Figure 6 of main text), which was obtained by calculating the electrostatic potential of the monolayer semiconductor in vacuum through the HSE06 level of DFT calculation with spin orbit coupling. Second, limited by the computational resource, the band gap of the MoS_2_ monolayer was aligned to the aqueous solution at the PBE level (Figure S4). The result was obtained by averaging 8 configurations obtained from the MD trajectory. The absorption spectrum was calculated through the SAS method at the HSE06 level of DFT calculation.

## 4. Conclusions

In conclusion, our analysis indicated that:

1. Water molecules adsorbed on TMDCs surfaces formed high-density water layers, with differently structured patterns of H-bonded water molecules (~4 H-bonds per water molecule) on different TMDC materials. These patterns were indicative of the hydrophobic character of the TMDC surface;
2. Some water molecules in the ad-layer were connected to bulk water through H-bonds, while some other waters had hydrogen atoms oriented towards the TMDC surface. This could facilitate proton transfer from bulk water to the TMDC surface via the Grotthuss mechanism in electrochemical or photocatalytic applications;
3. Despite the general trends described above, the ad-layers on top of the two TMDCs examined were different. The topology on top of MoS_2_ was denser, with a structure presenting four to six-water rings; while the topology on top of WSe_2_ had a higher tendency to form six-water rings;
4. The metal chalcogenide bonds of the TMDCs fluctuate up to ±5% from the equilibrium value during dynamics. Due to the anisotropic band gap dependence on deformations, the effects of stochastic deformation during the DFT-MD simulations tend to compensate each other. As a result, the band gap of MoS_2_ decreased by up to 0.2 eV. A smaller impact, 0.1 eV, was found on the band gap of WSe_2_. The impact of the TMDC structural deformations on the band alignment to vacuum was minimal;
5. The presence of explicit water molecules had similar negligible effect, below 0.05 eV, on the band gap and band alignment of the TMDCs. This result was consistent with the hydrophobic character and the apolar nature of infinite TMDC monolayers. Analysis of frames from the DFT-MD simulations evidenced a small effect of water and dynamics on the light absorption spectra, limited to a somewhat weaker absorption at shorter wavelengths and formation of a shoulder at longer wavelengths.

In summary, our study indicated that accurate modeling of monolayer TMDC materials for electrochemical/photocatalytic applications also requires consideration of explicit water molecules under dynamic conditions. Finally, our study also establishes a practical protocol for further exploration of water interacting with other (monolayer) semiconductors and their effects on material properties. Such protocol opens the opportunity to study a variety of scenarios, e.g., the presence of a second metal, or aqueous environment at different pH.

## Data Availability

The data that supports the findings of this study are available within the article and its Supplementary Material.

## Supplementary Materials

The following supporting information can be downloaded at: https://www.mdpi.com/article/10.3390/ijms231911926/s1.
