# Supplementary material for: Structural and Electronic Effects at the Interface between Transition Metal Dichalcogenide Monolayers (MoS2, WSe2, and Their Lateral Heterojunctions) and Liquid Water
Source: Int J Mol Sci. 2022 Oct 7;23(19):11926. doi: 10.3390/ijms231911926 (PMC9569863; doi:10.3390/ijms231911926)
Supplement: Supplementary file 1 [file ijms-23-11926-s001.zip › ijms-1941714-supplementary.pdf]

Supporting information for:

Structural and Electronic Effects at the Interface between Transition Metal Dichalcogenide Monolayers (MoS_2, WSe_2, and their Lateral Heterojunctions) and Liquid Water

By

Zhen Cao[1,*], Moussab Harb[1], Sergey M. Kozlov[2], Luigi Cavallo[1,*]

King Abdullah University of Science and Technology (KAUST), Physical Sciences and Engineering Division (PSE), Kaust Catalysis Center (KCC), Thuwal 23955-6900, Saudi Arabia.

2) Department of Chemical and Biomolecular Engineering, Faculty of Engineering, National University of Singapore, Singapore 119260

Table of contents

Schematic view of the armchair and zig-zag types of MoS_2-WSe_2 LHJs S3

Schematic view of scanning water on MoS_2-WSe_2 LHJs S4

Empirical force field parameters fitted from the DFT calculations S5

Snapshot of classical MD simulation and density profiles of the systems S6

Density profiles of water on TMDCs S7

Water probability maps on armchair and zig-zag types of LHJs S8

Hydrogen bonding networks on armchair and zig-zag types of LHJs S9

Water orientation map on armchair and zig-zag types of LHJs S10

Energy map of stressed MoS_2 and WSe_2 monolayer S11

Band alignment of MoS_2 monolayer toward the bulk water S12

Calculated light absorption of TMDCs and structures from MD simulations S13

Statistical analysis methods of water molecules at the interfacial region S14

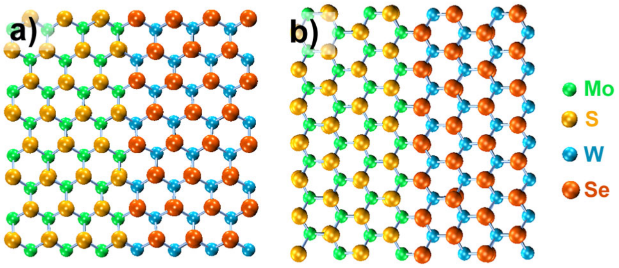

Figure S1. (a,b) Schematic view of MoS_2-WSe_2 armchair and zig-zag prototypical lateral heterojunctions; the figure also demonstrates the size of the TMDC supercells employed in this study.

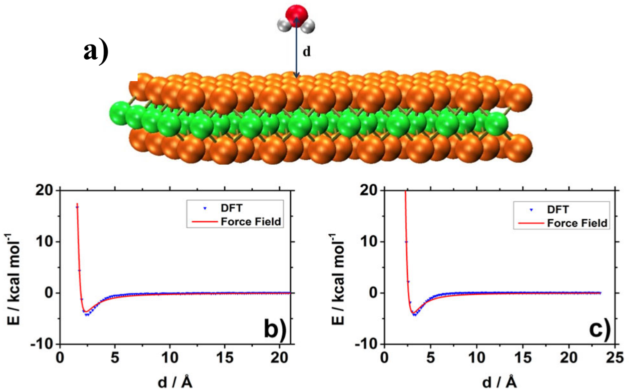

Figure S2. (a) Schematic view of the fitting scheme: the water-TMDC interaction energy was scanned as a function of distance between water and TMDCs surface. The comparison between the interaction energy calculated using the empirical parameter and DFT are shown in (b) for water-MoS_2 monolayer, and (c) water-WSe_2 monolayer, respectively.

Table S1. Empirical Parameters for water-TMDCs slabs fitted from the DFT calculations.

Atom Type	Partial Charge	Pairwise Interaction	ε_{ij}	Pairwise Interaction	σ_{ij}	Pairwise Interaction	ε_{ij}	Pairwise Interaction	σ_{ij}
**Mo**	0.397	**Mo-O**	0.864821	**Mo-O**	3.688089	**Mo-H**	0.0	**Mo-H**	/
**S**	-0.1985	**S-O**	0.17715	**S-O**	3.465902	**S-H**	0.0	**S-H**	/
**W**	0.274	**W-O**	0.657852	**W-O**	3.963807	**W-H**	0.0	**W-H**	/
**Se**	-0.137	**Se-O**	0.249465	**Se-O**	3.752706	**Se-H**	0.0	**Se-H**	/

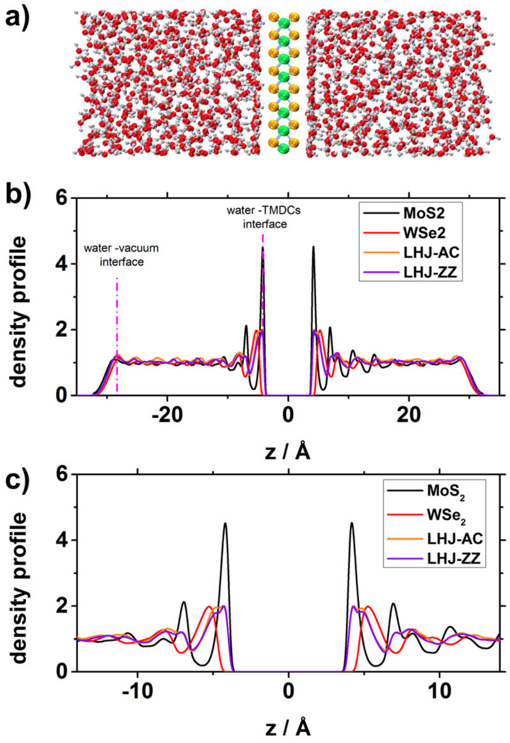

Figure S3. (a) Schematic view of large-scale classical MD simulation of TMDC with water. (b) Density profile of TMDC with water obtained from classical MD simulation trajectory: both water-vacuum interfaces and water-TMDCs interfaces are marked. The water-vacuum interface is to help water relax to its bulk density without artificial counting. Both water-vacuum interface (water-hydrophobic interface) and water-TMDCs interface can induce fluctuations reflected as peaks in density profiles. (c) Zoom in of the density profile in (b), demonstrating the water density can relax to 1 (bulk density).

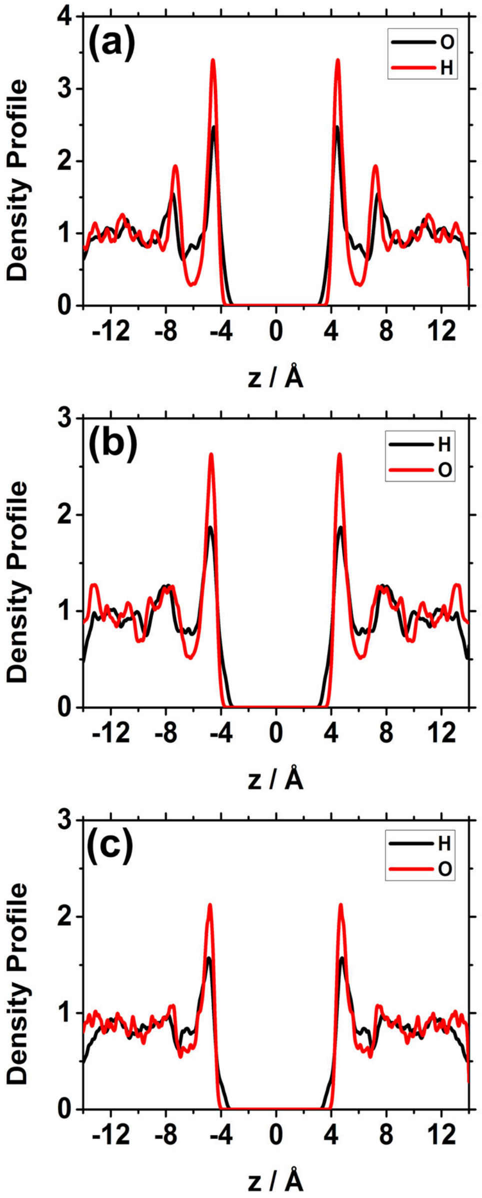

Figure S4. Density profile of hydrogen (black line) and oxygen (red line) of water surrounding monolayer MoS_2 (a), WSe_2 (b), and later heterojunction (c): The first peak is corresponding to the adsorbed water layer, and hydrogen shows a broader peak compared with that of oxygen atoms, indicating a variety of orientations of OH bonds in this layer; in the near-surface region, the density profile for hydrogen is above zero while it is zero for oxygen, indicating hydrogen can be closer to the 2D material surface.

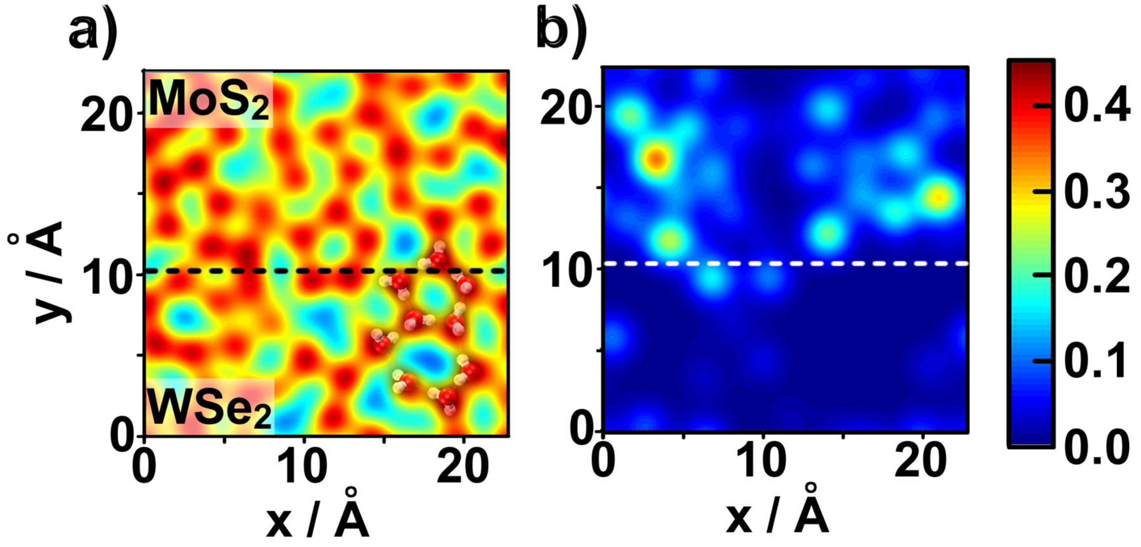

Figure S5. Probability maps, ϕw(x,y), for finding ad-layer water molecules on top (a) the armchair LHJ. Ad-layer water molecules correspond to water molecules within the first minima ($|z| \approx 5$ Å) of the water density profiles $\rho_O(z)$, shown in Figure 1. The map on panel (b) is obtained from the map on panel (a) by considering an ad-layer 15% thinner.

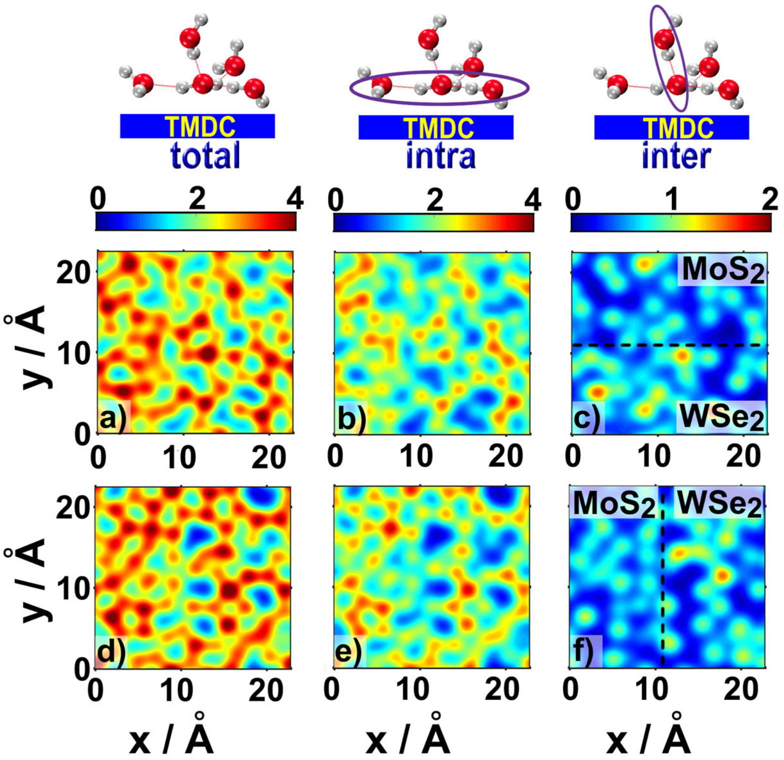

Figure S6. Hydrogen bonding network above TMDCs were evaluated in three ways: the total number of H-bonds for ad-layer water molecules, $n_{HB}(x,y)$, above armchair LHJ (a), and zig-zag LHJ (d). The total number of H-bonds were decomposed into H-bonds form by waters within the ad-layer (b), (e), and H-bonds between ad-layer water molecules and water molecules in above layer (c), (f).

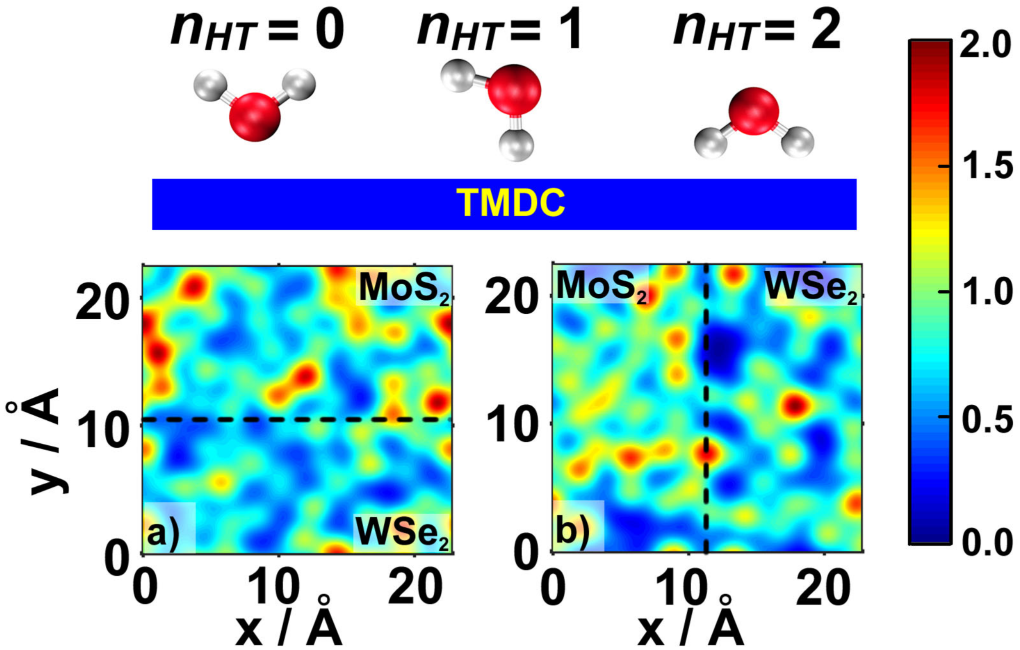

Figure S7. Orientation of water molecules above armchair (a) and zig-zag (b) types of LHJs.

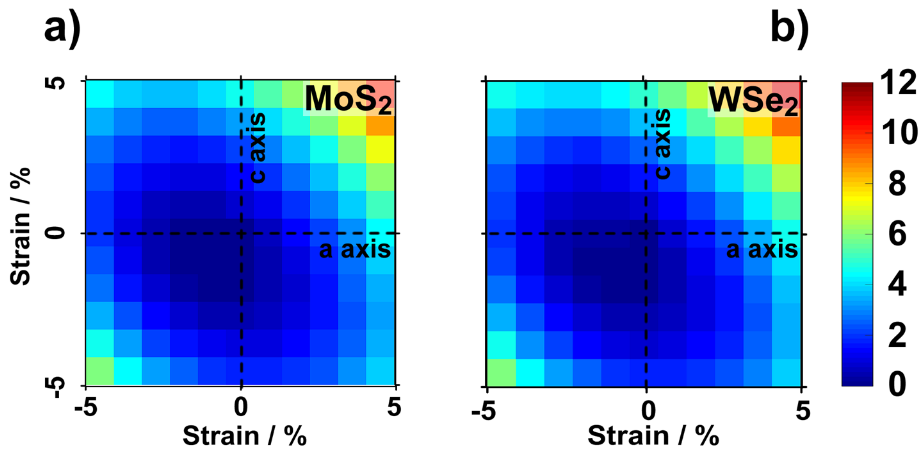

Figure S8. Energy map corresponding to the 5% strain of MoS2 (a) and WSe2 (b).

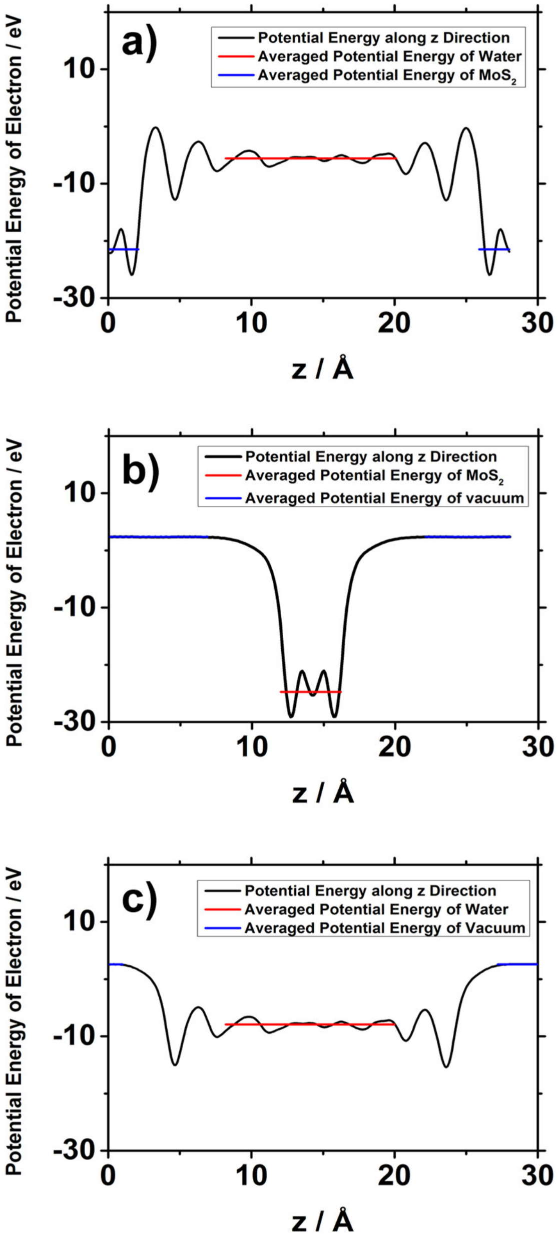

Figure S9. Schematic view of electrostatic potential energy presented at different conditions: (a) represents the TMDC monolayer in water; (b) represents the TMDC in vacuum; (c) represents the water in vacuum. The energy difference for different references indicates the band alignment can be achieved through: $\Delta E_{TMDC}^{water} = \Delta E_{TMDC}^{vacuum} - \Delta E_{water}^{vacuum} + \Delta E_{sol}^{eff}$.

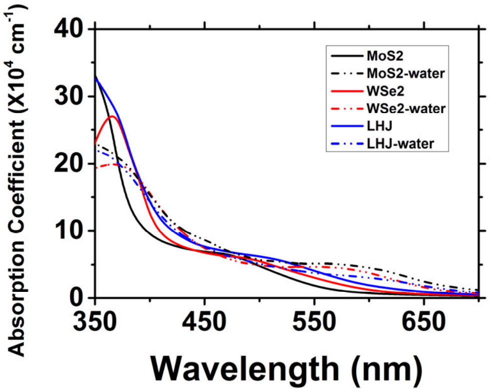

Figure S10. Representative solar light absorption spectra for MoS_2, WSe_2, and LHJs for both optimized structure (solid lines) and structure obtained from the DFT-MD trajectory (dashed lines).

Structural characterization of the interfacial water molecules.

1. Density profiles of the water molecules from the TMDC surface:

The density profiles of the water oxygen atoms [illegible](O) from the TMDC surface was characterized for water on MoS_2, WSe_2, and Armchair and Zigzag types of laterial heterojunctions, respectively. These density profiles were weighted by the bulk water density to demonstrate the liquid water at ~14 Å exhibits bulk water property.

2. Instantaneous liquid surface of the ad-layer water molecules:

To visualize water fluctuations in the ad-layer, the instantaneous liquid surface, as defined by Willard and Chandler is inserted in Figure 2 as an isosurface of Eq. 1,

$$\phi(r,t) = \sum_{i=1}^{N_{mol}} \frac{1}{\sqrt{2\xi^2\pi}} \exp\left(-\frac{\left(\vec{r}_i(t)-\vec{r}\right)^2}{2\xi^2}\right); \quad (1)$$

where $\vec{r}_i(t)$ is the position of the i-th water oxygen at time t, ξ = 2 Å determines the resolution of the grid used to build the coarse-grained interface.

3. Locations of the water molecules within the ad-layer

The structure and mobility of water molecules within the ad-layer is characterized using the water density $\varphi(\vec{a})$, as defined in Eq. 2,

$$\varphi(\vec{a}) = \left\langle \sum_{i=1}^{N_{mol}} \frac{1}{\sqrt{2\xi^2\pi}} \exp\left(-\frac{\left(\vec{a}_i(t)-\vec{a}\right)^2}{2\xi^2}\right) H\left(z_c - z_i(t)\right) \right\rangle; (2)$$

where $\vec{a}_i$ is the instantaneous position of the ith water oxygen projected onto the TMDC surface, H is the Heaviside step function, which is 1 if the ith water molecule is within the adsorbed layer at time t, i.e. $z_i(t) < z_c$, or 0 otherwise., $\langle\ \rangle$ indicates averaging over the last 4 ps of the DFT-MD trajectory. Here and further, ξ was set to be 1 Å for higher resolution. In turn, the cutoff distance z_c was set to the first

minima, d, of the water density profiles shown in Figure 2, except 0.85*d in Figure 3d.

4. Hydrogen bonding network (HBN) of the water molecules within the ad-layer

To characterize the obtained HBN structures, we plot $\psi(\vec{a})$ defined in Eq. 3, which provides a tendency of water molecules in the ad-layer to be engaged in H-bonds:

$$\psi(\vec{a}) = \left\langle \sum_{i=1}^{N_{mol}} \frac{N_i^{HB}(t)}{\sqrt{2\xi^2\pi}} \exp\left(-\frac{(\vec{a}_i(t) - \vec{a})^2}{2\xi^2} \right) H(z_c - z_i(t)) \right\rangle; \qquad (3)$$

where the pre-factor, $N_i^{HB}(t)$, is the number of hydrogen bonds formed by the ith water molecule at time t. The other terms are defined as in Eq. 2.

5. Detailed orientations of the water molecules within the ad-layer

To investigate the molecular orientation of adsorbed water molecules we plotted $\rho(\vec{a})$ of Eq. 4, which provides a propensity of waters in the ad-layer to interact with the TMDC via H atoms.

$$\rho(\vec{a}) = \left\langle \sum_{i=1}^{N_{mol}} \frac{n_i(t)}{\sqrt{2\xi^2\pi}} \exp\left(-\frac{(\vec{a}_i(t) - \vec{a})^2}{2\xi^2} \right) H(z_c - z_i(t)) \right\rangle; \qquad (4)$$

where $n_i(t)$ is the number of hydrogen atoms of the ith water molecule staying closer to the TMDCs surface than the oxygen atom in the same water
